# A 50-year journey in the development of treatment for benign prostatic hyperplasia

**DOI:** 10.1038/s41514-025-00231-2

**Published:** 2025-05-23

**Authors:** Andrew V. Schally, George Theodoropoulos, Wei Sha, Irving Vidaurre, Medhi Wangpaichitr

**Affiliations:** 1 Endocrine and Polypeptide Institute, Veterans Affairs Healthcare System, Miami, FL USA; 2 Department of Veterans Affairs, Miami VA Healthcare System, Research Service, Miami, FL USA; 3 South Florida VA Foundation for Research and Education, Veterans Affairs Healthcare System, Miami, FL USA; 4 Interdisciplinary Stem Cell Institute, University of Miami Miller School of Medicine, Miami, FL USA; 5 Department of Medicine, Divisions of Oncology and Endocrinology, University of Miami Miller School of Medicine, Miami, FL USA; 6 Department of Pathology, University of Miami Miller School of Medicine, Miami, FL USA; 7 Sylvester Comprehensive Cancer Center, University of Miami Miller School of Medicine, Miami, FL USA; 8 Department of Surgery, Division of Thoracic Surgery, University of Miami Miller School of Medicine, Miami, FL USA

**Keywords:** Drug discovery, Medical research

## Abstract

Recent research underscores the crucial role of hormone regulation in benign prostatic hyperplasia (BPH) and the therapeutic promise of growth hormone-releasing hormone (GH-RH) antagonists. BPH incidence in aging men doubled over three decades, driven by prostatic enlargement and lower urinary tract symptoms (LUTS). Aging-related changes in GH-RH and luteinizing hormone-releasing hormone (LH-RH) biology promote BPH through hormonal and inflammatory processes. Traditional therapies provide symptomatic relief but often fail to prevent progression. This review explores the 50-year extensive development of LH-RH and GH-RH peptide analogs from discovery to delivery and their potential in BPH treatment. In preclinical studies, GH-RH antagonists reduced prostate volume, improved LUTS, and modulated inflammation mediated by NF-κB and IGF-I. Clinical trials are needed to validate antagonist efficacy and safety. Given BPH’s public health impact among the aged, and especially among aging Veterans, integrating GH-RH antagonists into management strategies may offer precision-based therapeutic advancements.

## Introduction

Benign prostatic hyperplasia (BPH) is a progressive enlargement of prostatic glandular and stromal tissues, leading to lower urinary tract symptoms (LUTS) that significantly impact quality of life, particularly among the aged. BPH prevalence increases with age, affecting ~20% of men in their 40 s and up to 70% of men in their 60 s. Over the past three decades (1990–2021), the global incidence of BPH has risen dramatically from ~6.5 million to 13.5 million new cases annually, underscoring the urgent need for effective treatment options (Box 1).

Current medical therapies, including adrenergic blockers (e.g., terazosin) and 5α-reductase inhibitors (e.g., finasteride), provide symptom relief but do not fully address the disease’s underlying pathophysiology. For patients with severe BPH, surgical intervention, primarily transurethral resection of the prostate (TURP), remains a standard approach. However, the persistence of treatment failures and the progressive nature of BPH highlight the need for novel therapeutic strategies.

There is substantial evidence supporting the hormone-dependent nature of BPH during aging. Luteinizing hormone-releasing hormone (LH-RH) and growth hormone-releasing hormone (GH-RH) are critical in regulating growth hormone (GH) secretion and have been implicated in aging-related processes. Aging is associated with a decline in GH secretion, leading to reduced anabolic effects, increased adiposity, and decreased muscle mass. This hormonal shift contributes to frailty, metabolic dysfunction, and a decline in overall physiological resilience. In addition, aging-related changes in LH-RH and GH-RH signaling may influence inflammatory processes and the progression of age-related diseases, including BPH.

Experimental and clinical studies indicate that GH-RH and LH-RH analogs play a crucial role in prostate growth regulation. Prostate cells express receptors for these hypothalamic hormones, which can either stimulate hyperplasia or be blocked by antagonists to reduce prostate size and inflammation. Recent research has demonstrated that GH-RH antagonists not only shrink prostate volume but also exert potent anti-inflammatory effects, making them promising candidates for BPH treatment.

Overall, aging contributes to BPH by altering hormonal balance, increasing inflammation, and promoting prostatic tissue remodeling. This review provides a historical and scientific perspective on the evolution of hormonal therapies for BPH, focusing on the development of LH-RH and GH-RH antagonists. We discuss their molecular mechanisms, therapeutic potential, and the need for further clinical studies to optimize their application in BPH management.

Box 1 Increasing incidents of BPH from 1990 to 2021 The straight line represents the best linear fit to the data, and the purple band around the line shows the uncertainty in the linear fit.

The gray band represents a 95% confidence level. Data source: GBD 2021.
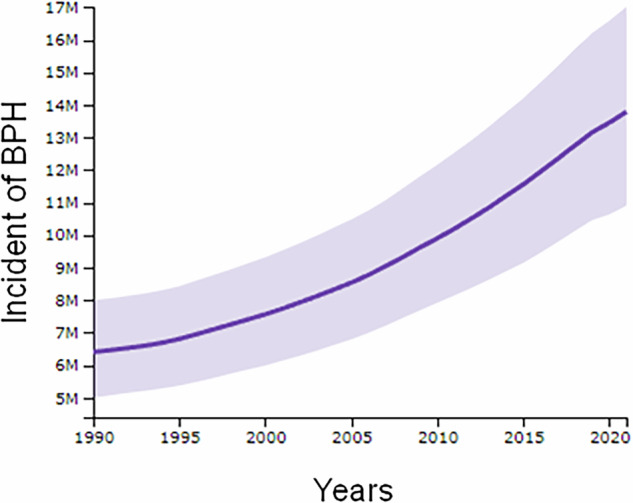

### Historical background and development of LH-RH analogs

**Fig. 1:**
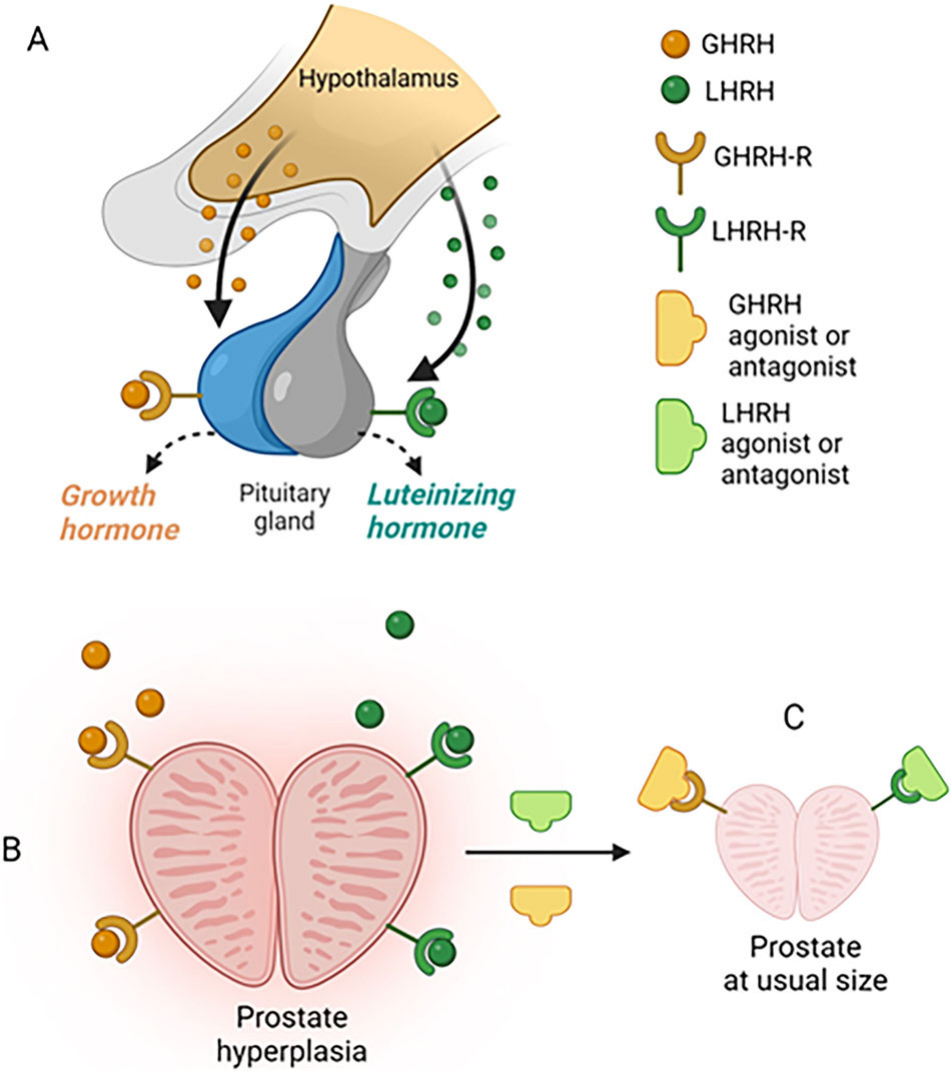
Impact of GH-RH and LH-RH on the development of BPH. **A** GH-RH and LH-RH are secreted by the hypothalamus, binding to pituitary receptors and stimulating GH and LH secretion. **B** Prostate cells express GH-RH and LH-RH receptors; thus, GH-RH and LH-RH can stimulate prostate hyperplasia. **C** Treatment with GH-RH agonists/antagonists or LH-RH agonists/antagonists can lead to the reduction of prostate size and inflammation.

The hypothalamus secretes GH-RH and LH-RH, binds to receptors in the pituitary receptors, and stimulates GH and LH secretion. In turn, GH-RH and LH-RH receptors expressed on prostate cells can be affected to stimulate prostate hyperplasia or blocked by agonists/antagonists (Fig. 1). Next, we will review the development of the agonistic and antagonistic analogs of LH-RH and GH-RH, concentrating on their applications related to therapy for prostate cancer and BPH itself.

The studies of G.W. Harris, a brilliant English physiologist, and others provided anatomic and physiological evidence in the late 1940’s^1^ that the hypothalamic portion of the brain controls the secretion of gonadotropins, luteinizing hormone (LH), and the follicle-stimulating hormone (FSH) from the anterior pituitary gland^1^. Some 20 years later, my group of investigators at the VA hospital and Tulane University School of Medicine in New Orleans purified porcine hypothalamic LH-RH, which unequivocally stimulated the secretion of LH and FSH in men and women in studies at the National Institutes of Nutrition in Mexico City^2^. My group then isolated LH-RH in pure form, identified its structure, synthesized it, and carried out extensive experimental and collaborative clinical studies^3^. This work and contributions by other investigators over the last 50 years (Box 2) showed that the LH- and FSH-releasing factor, often called Gonadotropin Releasing Hormone (Gn-RH) by me and others, is the main link between the brain and the pituitary for the control of reproductive functions and the central regulator of reproduction.

Box 2 Pioneering research and discoveries The timeline illustrates the history of key discoveries made in the field of hormone-releasing hormones.
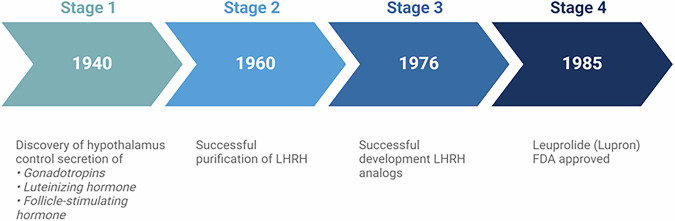

## LH-RH analogs

### Agonists

#### Agonistic analogs of LH-RH

There are more than 23 moieties of LH-RH among species, the mammalian structure pGlu–His–Trp–Ser–Tyr–Gly–Leu–Arg–Pro–Gly–NH2 being designated LH-RH type I (LH-RH I)^3^. The half-life of LH-RH is very short in plasma (about 2–4 min); thus, the development of longer-acting analogs for effective clinical applications is necessary. We predicted correctly in 1971 that replacing some amino acids would lead to analogs with increased or even antagonistic activity^3^. Substitution in positions 6 and 10 leads to highly active peptides. The first analog of LH-RH was [desGly10] LH-RH ethylamide, synthesized by Fujino et al.^4^, which was 3–5 times more active than LH-RH. Our group then synthesized analogs with d-amino substitutions like D-Leu6-LH-RH, which are 5-9 times more potent, and D-Trp6-LH-RH, which were even more active^3^.

Another series of analogs, like [d-Leu6-desGly10] ethylamide incorporated changes in both 6 and 10 positions, were 60 times more active in assays on LH release in rats or on ovulation^3^. The increased activity can be attributed to improved binding affinity to pituitary receptors, enhanced resistance to inactivation, and a conformational change induced by type β II bending. LH-RH analogs with substitutions in positions 6, 10, or both are much more active than LH-RH with prolonged activity^3^. Several analogs with substitutes in positions 6 and 10 or both have been used clinically.

#### Receptors for LH-RH

The effects of LH-RH on the secretion of FSH and LH are mediated by receptors for LH-RH expressed on membranes of the pituitary gonadotrophs^3,5^. The LH-RH type I receptor consists of seven α-helical transmembrane domains and an extracellular domain^5^. This receptor is coupled to the Gαq/11-protein. The receptors for LH-RH-I are also expressed in tumors such as breast, prostatic, and non-reproductive tissues, including glioblastoma^3^. Direct effects of LH-RH agonists and antagonists on the tumor cells are mediated by LH-RH-I receptors.

The binding of LH-RH-I to its pituitary receptors induces a micro-aggregation of receptors, and the complex formed is internalized and degraded^3,5^. In the pituitary, Gaq/11 proteins activate phospholipase C, stimulating the production of inositol phosphates and diacylglycerol. This leads to mobilization of Ca+ and activation of protein kinase C, producing secretion of LH and FSH^5^.

Different signaling mechanisms are involved in the actions of LH-RH-I- receptors on tumors. After binding of LH-RH, the LH-RH receptor-LH-RH complex is coupled to G α1 protein and activates phosphotyrosine phosphatase, which dephosphorylates receptors for epidermal growth factor (EGF), blocking the signal pathways^3,6^.

#### Antifertility effects of LH-RH agonists

Acute administration of LH-RH or its agonists induces a release of LH and FSH^2,3^. However, sustained administration of LH-RH or its agonists produces strong inhibitory effects that lead to the impairment of reproductive functions^3^. In normal men, chronic administration of LH-RH agonists produces a fall in the level of serum LH and FSH as well as testosterone levels^3^.

In female animals, continuous administration of agonistic analogs of LH-RH induces suppressive effects on the gonads. In women, chronic administration of high doses of LH-RH agonists suppresses levels of LH and ovarian estrogen. The inhibition of LH and ovarian steroidogenesis (steroid production) makes the therapeutic use of LH-RH agonists possible in endometriosis, uterine leiomyomas, and hirsutism^3^.

A sustained stimulation of the pituitary on repeated injections of LH-RH agonists or periodic administration of microcapsules (depot preparations) of LH-RH agonists suppresses the hypophyseal-gonadal axis through a reduction in pituitary receptors for LH-RH. The downregulation of LH-RH receptors creates a state of reversible medical castration.

The phenomena of downregulation of LH-RH receptors by LH-RH agonists have important critical applications^3^. For instance, in treating central precocious puberty and using LH-RH agonists in the in vitro fertilization and embryo transfer (IVF-ET), also known as controlled ovarian stimulation/assisted reproductive technology (COS-ART). This process relies on suppressing LH and FSH secretion from the pituitary, and it is classified as selective medical hypophysectomy. The treatment of sex hormone-dependent cancers, including prostate, breast, and endometrial cancers, relies on reversible medical castration to induce a state of sex steroid deprivation. This approach is also utilized for managing conditions such as uterine leiomyomas, endometriosis, and benign prostatic hyperplasia (BPH)^3^. LH-RH agonists not only suppress pituitary function but also exert direct inhibitory effects on multiple cancers, including breast, ovarian, endometrial, and prostate cancers. These effects are mediated through LH-RH receptors expressed on tumor cells, highlighting their role in targeted cancer therapy.

#### Use of LH-RH agonists for the treatment of prostate cancer

The greatest therapeutic benefits of LH-RH analogs were found in the field of prostate cancer^7^. Carcinoma of the prostate is the most frequent non-cutaneous malignancy and the second leading cause of cancer-related deaths among U.S. men^7^ with ~70% of prostate cancers being testosterone-dependent^8^. The treatment of hormone-sensitive prostate cancer introduced by Charles Huggins is based upon androgen dependence of the tumor and includes bilateral orchiectomy and administration of estrogens, but is linked to adverse side effects^9^.

In the early 1980s, our experimental work allowed me to introduce a new method for the treatment of advanced prostate cancer^3^ based on using LH-RH agonists to downregulate pituitary LH-RH receptors. This led to the inhibition of the secretion of LH and FSH from the pituitary gland and the suppression of testosterone and PSA levels in the blood^3^.

We were able to demonstrate that chronic administration of our LH-RH agonist [d-Trp6]-LH-RH could inhibit prostate tumor growth in two different rat models^10^. Thus, [d-Trp6]-LH-RH given at 25 µg/day for 3 weeks significantly inhibited the growth of chemically induced squamous cell carcinoma in Fisher male rats and significantly decreased the weights of the ventral prostate and testes. Treatment of male Copenhagen rats bearing the Dunning prostate (R-3327H) adenocarcinoma with 25 µg/day of d-Trp6-LH-RH for 6 weeks reduced tumor volume and decreased tumor weight^10^. In both rat models, serum testosterone levels were significantly reduced after treatment with [d-Trp6]-LH-RH. This study demonstrated the potential clinical efficacy of [d-Trp6]-LH-RH in the treatment of prostate carcinoma in man^10^.

A subsequent study^11^ in rats with Dunning R-3327H prostate tumors confirmed these findings. Treatment with [d-Trp6]-LH-RH for 3 weeks caused a highly significant reduction in tumor weight as compared with controls. Administration of [d-Trp6]-LH-RH significantly reduced LH levels and diminished serum testosterone values by 97%. These studies clearly demonstrated that chronic administration of the agonist [d-Trp6]-LH-RH inhibits the growth of Dunning prostate adenocarcinoma, a well-documented androgen-dependent prostate cancer^10,11^. These basic studies led to future clinical trials^12^.

The therapeutic efficacy of agonistic analogs of LH-RH in men with advanced prostate cancer was first demonstrated in collaboration with George Tolls et al. in a clinical trial at the Royal Victoria Hospital in Montreal in 1980-1981^12^. In this trial, ten patients with stage C prostatic carcinoma were treated with two agonistic analogs of LH-RH. [d-Trp6]-LH-RH given subcutaneously (s.c.) and [d-Ser(But)6] Des–Gly–NH210-LH-RH ethylamide also (s.c.) or intranasally. Plasma testosterone levels declined 75% by the third week of treatment. A decrease of plasma acid phosphatase and serum alkaline phosphatase occurred by the 10th week in patients with stage C disease presenting with prostatism, showing a major clinical improvement. In patients with Stage D disease and diffuse bone metastases, there was relief of bone pain documented by radioisotope bone imaging. It was concluded that agonistic analogs of LH-RH were most promising as therapeutic agents in patients with androgen-sensitive prostatic adenocarcinoma at that time^12^. This study was the first to demonstrate a fall in testosterone levels and marked subjective and objective improvement in patients with prostate carcinoma after treatment with agonists of LH-RH.

These findings have been confirmed and extended by many other clinical trials with LH-RH agonists in patients with prostate cancer in North America and Europe^7,13^. The Abbott group reported therapeutic trials at 13 U.S. centers with [d-Leu6]-LH-RH ethylamide (Leuprolide)^13^. They showed that the depot formulation of leuprolide is safe and effective in the treatment of advanced prostatic cancer, and that the safety and efficacy of this formulation did not differ significantly from those of the daily subcutaneous formulation.

In a clinical trial in France, 81 patients with stage B, C, and D prostatic carcinoma were treated daily subcutaneously for 3 months with the agonist [d-Trp6]-LH-RH (Decapeptyl)^14^. The findings were compared to those obtained with other hormonal therapies of prostatic carcinoma. Treatment with [d-Trp6]-LH-RH greatly reduced serum LH and testosterone levels. No flare of the disease occurred, and there were no side effects except for impotence. Treatment of prostate cancer based on LH-RH agonists averted the psychological impact of castration as well as the cardiovascular, hepatic, and mammary gland stimulation-related side effects of estrogen. Currently, the LH-RH analogs used clinically for the therapy of advanced prostate cancer include triptorelin (Decapeptyl, Trelstar)^14^, Leuprolide (Lupron, Eligard), buserelin (Suprefact, Suprecur) and goserelin (Zoladex).

#### Development of a sustained delivery for LH-RH analogs

Since daily injections of LH-RH analogs were inconvenient and occasionally produced compliance problems, in 1984 we developed a long-acting delivery system for the agonist [d-Trp6]-LH-RH in microcapsules of poly (DL-lactide-co-glycolide) (PLG) designed to release a controlled dose of the peptide over a 30-day period^15^. Other groups including ICI, (later AstraZeneca), Abbott- Takeda and Hoechst AG, (later Aventis) also developed depot formulations of their respective LH-RH agonists, goserelin, leuprolide, and buserelin consisting of microcapsules or implants in poly (lactide-glycolide) or other polymers administered once a month^3^.

The spherical microcapsules were formulated to release a dose of about l00 µg peptide per day over a 30–90-day period and were composed of 2–6% of the LH-RH analog dispersed in PLG polymer which is biodegradable and biocompatible. For the injection, the microcapsules are suspended in an aqueous injection vehicle containing carboxymethylcellulose or d-mannitol and Tween 20. Preparations of Lupron depot microspheres contain 3.75–7.5 mg leuprolide acetate and are injected i.m. Sustained delivery formulations of zoladex consist of cylindrical rods containing 3.6 mg goserelin and are administered s.c. as cylindrical rods of the polymer PLG31. Polyhydroxybutyrate tablets containing 3.6–5 mg of buserelin are implantable s.c.^3^. Improved depot preparations, which release leuprolide for 60–90 days or even up to 12 months and of triptorelin (Trelstar LA and Trelstar Depot) have also now been developed. Delivery systems for administration of LH-RH agonists to patients with prostate cancer are efficacious, convenient and also promote patient compliance. The acceptance of LH-RH analogs is excellent and LH-RH agonist therapy is the preferred method of treatment for men with advanced prostate cancer^3^.

## Antagonists

### Antagonistic analogs of LH-RH

In 1971, we also proposed that the replacement or deletion of some amino acids in the decapeptide sequence of LH-RH might result in analogs possessing structure requirement features for effective binding, but devoid of features necessary for a functional effect^16^. However, our proposal to synthesize a peptide that would inhibit the release of gonadotropins was initially met by much skepticism because, at that time, few effective inhibitors had already been developed. Nevertheless, given the growing interest in improving contraceptive methods, several research groups, including ours, have initiated systematic efforts to synthesize LH-RH antagonists. Our goal has been the development of more effective and reliable options. Since histidine (His) in position 2 and tryptophan (Trp) in position 3 appeared to have receptor binding and functional effects^16^. The early inhibitory analogs involved substitutions or deletions at these two positions. However, our findings revealed that analogs with a single deletion of His or Trp were not highly effective as antagonists. This insight prompted us to develop more potent analogs through additional structural modifications. The peptide sequence of [desHis2, Leu3, desGly10]-LH-RH ethylamide, [desHis2, d-Ala6, desGly10]-LH-RH ethylamide and [desHis2, Leu3, D-Ala6, desGly10]-LH-RH ethylamide caused an inhibition of LH-RH-induced release of LH and FSH in rats^17^. Replacing His in position 2 by d-Phe produced a better antagonist^18^. The D-Phe2- analogs were about three times more active than the corresponding desHis2-analogs. An assay based on the blockade of ovulation in cycling rats was used for testing antagonists^19^, and analogs such as [d-Phe2, d-Ala6]-LH-RH and [d-Phe2, d-Leu6]-LH-RH were able to block ovulation and the preovulatory surge of gonadotropin^19^. [d-Phe2, d-Phe6]-LH-RH was very effective in the rat, and modifying position 3 improved its inhibitory properties. The replacement of Trp by d-Trp in position 3 further increased the potency of inhibitory peptides. [d-Phe2, d-Trp3, d-Phe6]- LH-RH was very potent and was the first inhibitory analog active in human beings^3^. Since the replacement of Gly with basic d-Arg in position 6 caused the local anaphylactoid reaction, new analogs with neutral d-ureidoalkyl amino acids, such as d-citruline at position 6were synthesized by our group^20^. Among these antagonists free of edematogenic effects, [Ac-d-Nal(2)1, d-Phe(4Cl)2, d-Pal(3)3, d-Cit6, d-Ala10]LH-RH (SB-75), designated cetrorelix [Cetrotide], had high activity, and was synthesized first in our laboratory^20^ and later also made by Zentaris^21^.

Antagonists like antide (Nal-Lys antagonist); [N-Ac-d-Nal(2)1, d-Phe(4Cl)2, d-Pal(3)3, Lys(Nic)5, d-Lys(Nic)6, Lys(iPr)8, d-Ala10]LH-RH (Ares-Serono) and Nal-Glu antagonist [Ac- d-Nal(2)1, d-Phe(4Cl)2, d-Pal(3)3, Arg5, d-Glu6(AA), d-Ala10]LH-RH (NIH)^22^ were potent. Other antagonists that were developed in the field include; ganirelix [N-Ac-d-Nal(2)1, d-p-Cl- Antide Phe2, d-Pal(3)3, d-hArg(Et2)6, L-hArg(Et2)8, d-Ala10]LH-RH^23^ and degarelix [N- Ac-d-Nal(2)1, d-(p-Cl)-Phe2-d-Pal(3)3, 4Aph(L-Hor)5, d-4Aph(Cbm)6, Lys8(iPr), d-AlaI0]LH-RH (Ferring Pharmaceuticals)^24^.

Degarelix (Firmagon) is a decapeptide with 5 d-amino acids and two other non-coded amino acids. This is a third-generation LH-RH antagonist, developed by Ferring Pharmaceuticals for use in prostate cancer in the last decade and approved by the FDA for the treatment of prostate cancer^25^. Degarelix is soluble in aqueous solvents and given subcutaneously in forming gels at high concentrations and from which it can diffuse in a way similar to the release from depot formulations of peptide from microcapsules^25^.

LH-RH antagonists such as cetrorelix have major uses in gynecology, especially in IVF-ET/COS- ART, and seem to have various advantages over the agonists. Normal men showed a major fall in serum levels of LH, FSH, and testosterone a few hours after s.c. administration of 300 µg cetrorelix.

Antagonists of LH-RH show no biological stimulatory activity but compete with endogenous LH-RH for the LH-RH receptor sites producing a competitive blockade of LH-RH receptors^3^. Thus, antagonists cause a prompt inhibition of the release of gonadotropins and sex steroids in distinction to the LH-RH agonists. Therapy with antagonists also avoids a clinical flare in disease caused by a transient release of LH and sex steroids, which occurs in various patients during agonist administration. Our findings demonstrate that LH-RH antagonists induce downregulation of pituitary receptors for LH-RH and not only an occupancy of binding sites^26^.

### Experimental studies with antagonistic analogs of LH-RH in prostate cancer

The availability of sustained delivery systems consisting of microcapsules of the LH-RH antagonist cetrorelix made possible an investigation of their inhibitory effects on the growth of experimental prostate cancers. When a microcapsule preparation of cetrorelix was compared with microcapsules of agonist [d-Trp6]-LH-RH in rats bearing Dunning R-3327H prostate carcinoma, cetrorelix caused greater inhibition of prostate cancer growth than [d-Trp6]-LH-RH^27^, and decreased testosterone levels to non-detectable values, and LH levels were also diminished. Male mice bearing xenografts of PC-82 human prostate adenocarcinoma and receiving microgranules of cetrorelix had a greater decrease in tumor weight and volume than that produced by the microcapsules of the agonist^28^. The tumor inhibition induced by cetrorelix was demonstrated to be due to decreased cellular proliferation with enhanced apoptosis of the PC-82 tumors^28^. Testosterone serum levels were reduced by 94% by cetrorelix and levels of prostatic specific antigen (PSA) were significantly lower with the antagonist vs given agonists^28^. We also demonstrated that high doses of Cetrorelix inhibited growth of androgen-independent human DU-145 and PC-3 prostate cancers transplanted into nude mice, decreased the levels and mRNA expression of EGF receptors and of IGF-II in PC-3 and DU- 145 prostate cancers^29^.

Degarelix was also tested in various experimental models of prostate cancer and compared with [d-Trp6]-LH-RH, leuprolide and surgical castration. In the Dunning R-3327H rat carcinoma model, Degarelix induced a sustained inhibition of tumor growth^30^, but 40% of tumors recurred. In other preclinical studies, subcutaneous administration of degarelix produced a reduction in LH and testosterone in rhesus monkeys^31^. In animal models degarelix had lower activity for histamine release than the other LH-RH antagonists, ganirelix, abarelix, and cetrorelix^32^.

### Clinical studies with LH-RH antagonists in men with prostate cancer

Clinical trials showed persistent inhibition of testosterone levels in patients with advanced (stage C and D2) prostatic cancer treated bid s.c. with 500 µg cetrorelix for several months^33^. After 6 weeks, the levels of PSA, and serum acid and alkaline phosphatases fell to normal values^33^. Cetrorelix was also evaluated in five patients with advanced carcinoma of the prostate and paraplegia due to metastatic compression of spinal cord, who could not be treated with LH-RH agonists because of the risk of “flare”^34^. Cetrorelix was administered at doses of 500–800 µg b.i.d. for 3 months. Cetrorelix and related LH-RH antagonists such as degarelix could be useful for patients with prostate cancer and metastases in the brain, spine, liver, and bone marrow^34^.

Degarelix was evaluated for safety and effectiveness in three phase II clinical trials in Europe, South Africa, North America, and Japan. Open-label^3,24,35^, randomized studies that included only histologically confirmed prostate cancer. The European study randomized 189 patients to loading doses of 200 or 240 mg degarelix followed by monthly maintenance doses by s.c.^24^. The North American trial treated 127 patients: a loading dose of 200 mg degarelix followed by monthly maintenance of 60 or 80 mg^35^. In all three phase II trials, degarelix was well tolerated, and treatment was associated with the suppression of testosterone and PSA.

Based on these clinical dose-finding trials, a 1 year, randomized, phase III trial was conducted in North America and Europe^36^ including 610 patients with prostate cancer. Using degarelix loading dose of 240 mg for the first month, and maintenance doses of 80 or 160 mg was compared to leuprolide at 7.5 mg per month^36^. Degarelix groups were significantly more rapid in suppression of PSA compared to the agonist leuprolide group and produced a more rapid suppression of LH and FSH levels. Both these gonadotropins remained suppressed until the trial’s end. In the leuprolide arm of this study, however, there was an initial increase in LH and FSH, and FSH levels never fell as much as in the degarelix arms^36^.

## Early clinical research for BPH

The experimental effects in the prostate cancer model allowed us to grasp the effects in the animal model of BPH inhibition. In fact, the first studies on the treatment in men with BPH were clinical, not in lab experiments, and were based on agonistic analogs of LH-RH^37^. Peters and Walsh used nafarelin acetate, the agonist of LH-RH, in men with symptomatic BPH and found an improvement, but upon cessation of treatment, the BPH symptoms returned^37^. Gabrilove et al.^38^ also used another LH-RH agonist, d-Leu6, Pro9-NHEt-LH-RH (Lupron, Abbott-Takeda)^38^ for 4–6 months, reporting a reduction in prostate size and improvement in 15 men with BPH^38^. However, the effects of medical castration induced by agonistic LH-RH analogs (shrinkage of the prostate and improvement in urinary symptoms) were reversed when the therapy was discontinued^38^.

Better results on BPH were obtained by us using LH-RH antagonists. First, we demonstrated potent inhibition of secretion of gonadotropin, a sex steroid in mice, rats, men, and women by LH-RH antagonists Ac-d-Nal(2)1, d-Phe(4Cl)2, d-Pal(3)3, d-Cit6, d-Ala10-LH-RH (SB-75, cetrorelix)^39^. Then in the first BPH study, we treated 11 patients with BPH with cetrorelix (0.5 mg, twice daily, s.c.) for 4 weeks^33^. There was a rapid reduction in prostate volume or an increase in urinary flow rates, suppression of growth factors, improvement in international prostate symptom sexual function scores, and a decline in serum prostate-specific antigen (PSA)^33^. The study demonstrated that the treatment of BPH with cetrorelix was safe and led to long-term improvement^33^ (rapid symptomatic improvement of BPH sustained for 6 months).

### Cetrorelix

In the second study, we investigated the efficacy and safety of cetrorelix in the treatment of symptomatic BPH^40^. By investigating whether short-term administration of cetrorelix could provide an improved treatment for men with BPH. Thirteen patients with moderate to severe symptomatic BPH were treated with cetrorelix (5 mg, s.c., twice daily for 2 days followed by 1 mg/day, s.c., for 2 months) and evaluated at baseline, during treatment, and up to 18 months after therapy for effects on the International Prostate Symptom Score (IPSS), Quality of Life score, sexual function, prostate size, uroflowmetry, and hormonal levels. Cetrorelix produced a decline of 52.9% in IPSS, a 46% improvement in the QOL score, a rapid reduction of 27% in prostatic volume, and an increase in peak urinary flow rates by 2.86 mL/s^40^. Serum testosterone fell to castration levels on day 2 but was inhibited only by 64–70% during maintenance therapy, and returned to normal after cessation of treatment. During long-term follow-up, most patients continued to show a progressive improvement in urinary symptoms (decline in IPSS from 67% to 72% at weeks 20 and 85, respectively) and an enhancement of sexual function, and prostatic volume remained normal. Our studies demonstrated that in patients with symptomatic BPH, treatment with cetrorelix was safe and produced long-term improvement^40^.

Clinical studies and phase III trials on cetrorelix in patients randomized to cetrorelix vs. placebo^41^ followed the first two BPH studies^33,40^. However, studies were not properly conducted in many clinics, and except for top centers^41^ did not show significant differences between treatment groups. These findings and the other problems of the pharmaceutical company involved caused the temporary suspension of this clinical research on BPH. The mechanisms involved in the inhibition by the BPH antagonist, cetrorelix were studied in vitro^42^. Subsequent clinical studies revealed a potentially important role for inflammation in the development of BPH^43,44^. Results showed that infiltrating T cells and macrophages produced the cytokines IL-2 and INF-γ^43^, supporting fibromuscular growth. This was achieved via proinflammatory cytokines and growth factors like IGF-1 and lead to LUTS (problems with urination).

In view of finding that BPH had an inflammatory component and that therapy with cetrorelix caused an improvement in LUTS in men with symptomatic BPH, our group investigated the mechanism of action and effect of cetrorelix in a male Wistar rat model of BPH^45^. BPH was induced in rats by subcutaneous injections of Testosterone Enanthate (TE) 2 mg/day for 4 weeks, while control animals received injections of corn oil. After induction of BPH, rats received depot cetrorelix pamoate at doses of 0.625, 1.25, and 12.5 mg/kg on days 1 and 22, and control rats received vehicle. Prostate weights were significantly lowered by any dose of cetrorelix^45^. The hormones, cytokines, and growth factors were also significantly decreased (Table 1).

**Table 1:** Quantitative analysis of molecules significantly reduced by cetrorelix

Hormones and proteins	Inflammatory cytokines	Growth factors
DHT, LH, IGF-1, and PSA	IFN-g, IL-3, IL-4, IL-5, IL-6, IL-8, IL-13, IL-15, and IL-17	EGF, FGF-2, FGF-7, FGF-8, FGF-14, TGF-b1, and VEGF

This study showed that cetrorelix reduces various proinflammatory cytokines and growth factors in rat prostate and, at doses which do not induce castration levels of testosterone, can lower prostate weights. Our findings shed light on the mechanism of action of LH-RH antagonists in BPH^45^. The model of BPH in male rats produced by repeated injections of testosterone was adapted for several studies, including our own^45^. The classification of human BPH, being predominantly of epithelial origin, supports the use of a testosterone-induced model of BPH with predominant epithelial hyperplasia. Antagonists of growth hormone-releasing hormone (GH-RH) reduced prostate size in experimental BPH^46^, and we previously showed that potent synthetic antagonists of GH-RH strongly inhibit the growth of diverse experimental human tumors including prostate cancer by suppressing various tumoral growth factors^47,48^. Hence, the combination of the initial clinical studies, and the added molecular findings from animal studies provided some support for these agents having therapeutic potential in the clinic (Table 2).

**Table 2:** Clinical findings of LH-RH agonists/antagonists used in the treatment of BPH

Therapy	Purpose	Conclusions
Goserelin^97^	The potential of goserelin acetate in improving surgical outcomes for BPH patients undergoing transurethral resection of the prostate (TURP) by minimizing intraoperative bleeding.	Clinical trial with 68 BPH patients receiving a single injection of goserelin acetate four weeks before surgery. Patients treated with goserelin acetate experienced a shorter surgery duration, lower blood loss, and more tissue removal during TURP.
Degarelix (Trial# NCT0094 7882)	A trial of degarelix in men with LUTS associated with BPH. Aimed to determine the optimal dose of degarelix and its effects on prostate volume, quality of life, and testosterone suppression-related safety issues.	Clinical trial with 380 men with moderate to severe LUTS. Degarelix significantly improved LUTS by reducing prostate volume and improving urinary flow. Patients experienced reduced symptoms such as urgency, frequency, weak flow, and incomplete bladder emptying.
Cetrorelix^40^	Phase 3 trial assessed the efficacy and safety of cetrorelix in 667 patients with BPH.	Cetrorelix treatment resulted in significant improvements in urinary symptoms and quality of life over 8 weeks. High scores of urinary symptoms decreased significantly, indicating the potential in managing BPH.
Leuprolide^98^	Adding Leuprolide acetate to the standard treatment for BPH, including a 5-alpha reductase inhibitor and an alpha-1-adrenergic inhibitor in 77 patients.	A statistically significant improvement in the International Prostate Symptom Score (IPSS) in the intervention group vs. the control group. Clinical studies showed that leuprolide is effective in reducing prostate volume and improving symptoms such as urinary flow and retention.

### Degarelix

Various trials comparing Degarelix to LH-RH agonists, identified differences^49^ with the rate of progression, of PSA, cardiovascular (CV) event rate, and overall survival, significantly favoring Degarelix. Patients with preexisting CV disease had markedly less frequent occurrences of CV events. The results suggest, that Degarelix may protect against CV disease in men with prior disease. A study by Margel et al.^50^ showed that patients treated with Gn-RH agonists experienced significantly more major cardiovascular and cerebrovascular events than those treated with Gn-RH antagonists. These Phase II results suggest that in prostate cancer patients who have preexisting cardiovascular disease, selecting the androgen deprivation therapy modality may differentially affect cardiac outcome^49^. These findings may also suggest differential effects on FSH^24^ and/or endothelial plaques from degarelix, and need to be confirmed in a prospective study^49^. In summary, clinical trials illustrated long-term efficacy for degarelix similar to leuprolide in achieving suppression of testosterone in patients with prostate cancer^3^. Degarelix produces a faster suppression of testosterone and PSA with no microsurges of testosterone. Treatment with degarelix improved disease control as vs LH-RH agonist measured by PSA progression-free survival, and a better suppression of bone serum alkaline phosphatase and FSH^49^.

#### Relapse from androgen control

LH-RH agonists and antagonists provided effective palliative therapy in patients with advanced prostate carcinomas. However, all hormonal therapies aimed for androgen deprivation include orchiectomy and anti-androgens. In most cases, LH-RH analogs and their combination provided remissions of only a limited duration^51^. Intracrine androgen synthesis may have led to restored AR signaling, leading to a rise in PSA levels and recurrent tumor growth, a relapsed state commonly referred to as castration-resistant prostatic cancer (CRPC)^8^.

Another mechanism responsible for the relapse could be attributed to a selective proliferation of clones of androgen-independent cancer cells present within heterogeneous tumors. Testosterone-insensitive cells can proliferate, eventually becoming predominant. Most patients with advanced^52^ prostatic carcinoma relapse in 15–36 months depending on the study with clinical, radiological, or biochemical manifestation, the last by a rise in serum PSA. These patients usually die of androgen-independent CRPC.

Therapeutic options for those patients who relapse after androgen control are limited. Interfering with the signal transmission of IGF-I, EGF, or their receptors by antagonists of GH-RH could inhibit the growth of androgen-independent prostate conditions and delay relapse. Our findings strongly suggested that further studies of GH-RH antagonists could lead to methods for effective prostate treatment.

## GH-RH analogs

### New approaches to the treatment of BPH using analogs of GH-RH

In this article, we first reviewed the action and uses of agonists and antagonists of LH-RH, and we will now review the development of agonists and antagonists of GH-RH before focusing on their application as single or combined agents for the treatment of BPH. Our laboratory is well-known for pioneering work on LH-RH and its analogs and contributing to the development of their clinical uses for the past 3 decades^3^. Since 1995, we have been focusing on the development of agonists and antagonists of GH-RH^53^. Next, we describe our various early attempts to evaluate GH-RH analogs experimentally in models of BPH and even some preliminary clinical studies in selected patients suffering from BPH.

Human growth hormone-releasing hormone (hGH-RH) is a hypothalamic peptide hormone that regulates growth hormone (GH) secretion (production and release) from the anterior pituitary^54–56^ through its binding to pituitary GH-RH receptors/(GH-RH- R)^57^. As a 44-amino acid peptide hormone, hGH-RH belongs to a family that includes vasoactive intestinal peptide (VIP), secretin, glucagon, glucagon-like peptides-1 and -2, and gastric inhibitory peptide^54–58^.

GH-RH also regulates the insulin-like growth factor-I (IGF-I) by activating the pituitary GH/liver IGF-I axis^58^. GH-RH and its analogs exert direct effects on a variety of extra-pituitary cells/tissues, promote the survival of cardiomyocytes, protect rat heart, induce normoglycemia stimulate/ migration and proliferation of mouse embryonic fibroblasts (MEFs) in vitro, accelerate healing of skin wounds in vivo^59^. Thus, studies have shown multiple therapeutic roles for GH-RH^58^ and its agonists in a wide range of medical fields and indications^60^.

Our long-term goal was the development of GH-RH agonists with appropriate biological and pharmaceutical properties for use in clinical settings^61^. However, native GH-RH has a short in vivo half-life, a result of its degradation by proteolytic enzymes. Therefore, our laboratory developed the MZ-, JI-, and MR-series of GH-RH agonists with enhanced GH-releasing potency both in vitro and in vivo^53,62^.

## Agonist

### Development of agonistic analogs of GH-RH

The first synthetic analog we developed, [Nle27] GH-RH (1–29) NH2, replacing Met27 with norleucine (Nle), effectively stimulated GH secretion via intravenous and subcutaneous administration^63,64^. Further modifications in the MZ-series, such as replacing tyrosine with 2,4-Diaminobutyric acid (Dat) and arginine-NH2 with agmatine, increased enzymatic stability and potency^65^. The JI-series focused on preventing trypsin-mediated degradation, replacing lysine residues with ornithine (Orn) and alanine with α-aminobutanoyl (Abu). These changes led to JI-34, JI-36, and JI-38, which exhibited up to 116.8 times higher potency than native GH-RH^66^. A key advancement was the development of MZ-3-149, an analog that contained aspartic acid at position 28. We found that this modification allowed for more efficient pulmonary inhalation and subsequent delivery, demonstrating strong GH release and clinical potential. These analogs represent promising candidates for therapeutic applications.

### Effects of GH-RH agonists on tumor growth

In vitro, GH-RH agonist MR-409 promoted cell growth in cancer lines and reduced cell apoptosis^58^. Surprisingly, in vivo, the effects of GH-RH agonists on tumor growth turned out to be the opposite. Daily s.c. administration of agonist MR-409 to mice bearing xenografts of lung cancers significantly inhibited tumor growth after treatment of 4–8 weeks^67^. The inhibition of tumor growth in vivo by MR-409 seemed similar to that induced by GH-RH antagonists MIA-602 or MIA-690^47^. This blocking of tumor growth by agonists of GH-RH, which at first may seem paradoxical, appeared to be caused mainly by the downregulation of the pGHRH-Rs and tumoral GH-RH-Rs. These phenomena are thus analogous and correspond to the well-known downregulation of receptors for LH-RH^3^, induced by therapy with agonistic analogs of LH-RH^3^. This event is thoroughly documented clinically and leads to suppression of the levels of LH, FSH, and sex steroids and inhibition of the growth of prostatic and breast cancers, as reviewed in Schally et al.^3,68^. Thus, short-term activation of GH-RH receptors by GH-RH agonists in vitro leads to stimulatory effects, but chronic, long-term, persistent exposure to these ligands produces a downregulation of receptors and inhibitory effects^58,67^.

### Effects of GH-RH agonists on BPH

GH-RH agonists have been investigated in various non-prostatic tissues, where they exert anabolic and tissue-regenerative effects, suggesting that they may also play a role in prostate cell growth. As mentioned above, despite the theoretical possibility that GH-RH agonists could stimulate prostate cell proliferation through GH/IGF-1 signaling, in vivo studies have paradoxically shown inhibitory effects on tumor growth due to receptor downregulation. The effect of MR-409-associated inhibition of tumor growth in lung cancer models despite initial in vitro stimulation of cell proliferation was a phenomenon attributed to chronic receptor downregulation. The complexity of the GH-RH impacts on cell proliferation warrants further preclinical and clinical studies to determine whether GH-RH agonists might promote or inhibit BPH progression. At this time, the evidence suggests that GH-RH antagonists remain the more promising therapeutic approach for BPH, given their well-documented antiproliferative and anti-inflammatory effects.

## Antagonist

### Antagonistic analogs of GH-RH

In the past few decades, many antagonists of human GH-RH have been synthesized and tested by other investigators, as well as by us. Our laboratory developed several classes of GH-RH antagonists that show potent inhibitory effects on the growth of various tumors^61^. Strategies to improve bioavailability, half-life in vivo, rapid renal clearance of GH-RH antagonists, and in vivo stability were developed^48,65,69–72^.

We incorporated pentafluoro-Phe among the structure modifications at different positions into several GH-RH analogs. Acylation of GH-RH antagonists with octanoic acid or 12- aminododecanoic acid improved the antiproliferative effects of these antagonists. Our work between 1994 and 2022 resulted in several series of potent GH-RH antagonists intended for cancer therapy. We described the design and syntheses of a new class of GH-RH antagonists with greater tumor inhibitory potency and augmented suppression of the GH release^47,73^.

### Effects of GH-RH antagonists on tumor growth

We have shown that GH-RH antagonists effectively suppress tumor growth by blocking GH-RH receptors on cancer cells, thereby disrupting key signaling pathways involved in proliferation, survival, and metastasis. MIA antagonists suppressed tumor growth in microgram doses of subcutaneous administration of diverse human cancer lines xenografted into nude mice. Antagonists MIA-602 and MIA-690 were among the most potent anti-tumor analogs and also displayed anti-inflammatory activities^47^ powerfully hindering tumor growth. GH-RH antagonists of the MIA class inhibited tumor growth in vivo in nude mice of some 16 types of solid human cancers represented by nearly 50 human cancer lines. Western blot and qRT-PCR analyses demonstrated the downregulation of expression of the pituitary-type GH-RH-R and splice variant SV1^47^. Tumor inhibition was accompanied by the downregulation of the pituitary type and splice variant SV1 receptors in cancer cells^47^.

Recently, we also completed the syntheses and biological evaluation of GH-RH antagonists of the AVR series with higher anticancer activities compared to our GH-RH antagonists of the MIA series^47,70–72^. AVR analogs contained additional modifications at positions 0, 6, 8, 10, 11, 12, 20, 21, 29, and 30^73^ and presented better binding affinities to the membrane GH-RH receptors on the human pituitary than MIA-602. After subcutaneous administration at 5 µg/day doses, some AVR antagonists demonstrated better inhibition of tumor growth in nude mice bearing various human cancers, with analog AVR-353 inducing stronger suppression than MIA-602 in lung, gastric, pancreatic, and colorectal cancers and AVR-352 in ovarian cancers and glioblastoma^73^. AVR-352 also demonstrated higher anti-inflammatory effects in lung granulomas from mice with lung inflammation.

Earlier generations, including MZ-, JV-1-, and JMR-series antagonists, showed efficacy in endometrial^74^, renal^75^, lung^76–78^, glioblastoma^79^, breast^80^, and colon cancers^79^. Expanding upon these findings, MIA-602 has exhibited significant tumor suppression in esophageal^81^, gastric^82^, and thyroid cancers^83^, as well as pleural mesothelioma^84^, prostate cancer (both androgen-dependent and castrate-resistant forms)^85,86^, pituitary adenomas^87^, melanoma^88^, and leukemias^89^. Many of these disorders impact patients as they age, making the application of these antagonists pertinent to the aging population. A particularly promising aspect of GH-RH antagonists is their potential to enhance existing cancer therapies. When combined with chemotherapy or radiation, these antagonists amplify treatment effectiveness, resulting in greater tumor reduction and improved therapeutic response^90,91^. This ability to work synergistically highlights their potential as a novel approach in cancer treatment, especially in aggressive and treatment-resistant malignancies. Given their broad-spectrum anticancer activity and ability to modulate the tumor microenvironment, further clinical evaluation of GH-RH antagonists could lead to new therapeutic advancements in oncology.

### Effect of GH-RH antagonists on BPH

The influence of GH-RH antagonists on animal models of BPH had not been investigated hence, we evaluated the effects of the GH-RH antagonist JMR-132 given at doses of 40 μg/day, MIA-313 at 20 μg/day, and MIA-459 at 20 μg/day in testosterone-induced BPH in Wistar rats^46^. Reduction of prostate weights was observed after 6 weeks of treatment with GH-RH antagonists: 17.8% decrease with JMR-132; 17.0% decline with MIA-313; 21.4% reduction with MIA-459. We measured transcript levels of genes related to growth factors, inflammatory cytokines, and signal transduction and identified significant changes in the expression of more than 80 genes. Significant reductions in protein levels of IL-1β, NF-κβ/p65, and cyclooxygenase-2 (COX-2) were also observed after treatment with a GH-RH antagonist. We conclude that GH-RH antagonists can lower prostate weight in experimental BPH by the direct inhibitory effects of GH-RH antagonists exerted through prostatic GH-RH receptors. This study shed light on the mechanism of action of GH-RH antagonists in BPH, suggesting that GH-RH antagonists should be considered for further development as therapy for BPH^46^.

We then showed that combining antagonists of GH-RH with antagonists of LH-RH greatly augmented the shrinkage of BPH^92^. We evaluated the effects of the GH-RH antagonist JMR-132 (40 µg daily), the LH-RH antagonist cetrorelix (0.625 mg/kg), and their combination on testosterone-induced BPH in adult male Wistar rats in vivo^92^. Prostate tissue was examined with marked shrinkage of the rat prostate (30.3%) occurring in response to the combination of GH-RH and LH-RH (Gn-RH) antagonists. The combination strongly decreased prostatic prostate-specific antigen, 6-transmembrane epithelial antigen of the prostate, interleukin-1β, nuclear factor-Kβ and cyclooxygenase-2, and decreased serum prostate-specific antigen^92^.

We also showed mechanisms of synergism between antagonists of GH-RH and antagonists of LH-RH in shrinking experimental BPH^93^. Since the combination therapy of antagonists of GH-RH and of LH-RH (Gn-RH) induced prostate shrinkage in rat models, we investigated the mechanisms of action of this combination on cell cycle traverse and expression of prostatic genes. Effects of the GH-RH antagonist, JMR-132 (40 µg/day), the LH-RH antagonist, Cetrorelix (0.625 mg/kg), and their combination were evaluated on testosterone-induced BPH in male Wistar rats. Cell viability was assessed by MTS assay in BPH-1 human prostate epithelial cells and WPMY-1 normal prostate stromal cells and cell cycle was analyzed by laser flow cytometry. RT- PCR arrays were performed, and the combination of antagonists caused marked shrinkage of rat prostate (30%). In vitro, GH-RH antagonists JMR-132 plus cetrorelix at 5 µM produced synergistic (57.4%) inhibition of the growth of BPH-1 cells, and a slightly lesser inhibition (46%) of WPMY-1 cells. Co-treatment with JMR-132 plus cetrorelix induced a significant increase of BPH-1 cells blocked in S-phase plus cells with lower G0/G1 and G2/M DNA content. Significant changes in expression of >40 gene transcripts related to growth factors, inflammatory cytokines, and signal transduction were identified. In conclusion, the combination of GH-RH and LH-RH antagonists potentiated reduction of rat prostate weight and synergistically inhibits growth of BPH-1 cells leading to cell cycle arrest in S-phase^93^. These effects were lesser in normal stromal prostate cell line, WPMY-1. Our findings reinforce the view that GH-RH antagonists should be useful for BPH therapy, possibly in combination with LH-RH antagonists^93^.

Antagonists of GH-RH inhibit proliferation induced by inflammation in prostatic epithelial cells^94^. GH-RH was shown to act as a paracrine/autocrine factor in various malignancies, including prostate cancer. GH-RH and its receptors are expressed in experimental models of BPH, in which antagonists of GH-RH suppressed the levels of proinflammatory cytokines and altered the expression of genes related to epithelial-to-mesenchymal transition (EMT). We investigated the effects of GH-RH antagonists on prostatic enlargement induced by inflammation^94^. Autoimmune prostatitis in Balb/C mice was induced by a homogenate of the reproductive tissues of male rats. During the 8-week induction of chronic prostatitis, we detected a progressive increase in prostatic volume reaching 92% at week 8 compared with the control. Daily treatment for 1 mo. with GH-RH antagonist MIA-690 caused a 30% reduction in prostate volume. Conditioned medium derived from macrophages increased the average volume of spheres by 82.7% and elevated mRNA expression for N-cadherin, Snail, and GH-RH. GH-RH antagonist reduced the average volume of spheres stimulated by inflammation by 75.5%, and TGF-β2 by 91.8%^94^. The proliferation of primary epithelial cells stimulated by IL-17A or TGF-β2 was also inhibited by 124.1% and 69.9%, respectively^94^. GH-RH stimulated the growth of BPH-1 and primary prostate spheres. This study provided evidence that GH-RH plays important roles in prostatic inflammation and EMT, suggesting the merit of clinical investigation to elucidate the effects of GH-RH antagonists in prostatitis and BPH. These data also shed light on the paracrine roles of GH-RH in prostatic inflammation and demonstrated that GH-RH stimulated the growth of BPH-1 and primary prostate epithelial spheres^94^.

BPH is assumed to be the result of age-related processes, including age-dependent tissue remodeling and the increase in the ratio of estradiol/dihydrotestosterone. BPH enlargement of the prostate occurs due to an increase in the number of epithelial and stromal cells. Most intriguingly, inflammation has a major role in the etiology of BPH^45,93–95^. In addition, stromal cells from the aging prostate secrete increased levels of chemokines, which attract macrophages and T-lymphocytes. Macrophage-conditioned medium induces EMT transition in vitro, the formation of new acini, which may be a key process in the development of glandular BPH.

We reported that GH-RH is upregulated and serves as a local growth factor in animal models of testosterone-induced BPH. GH-RH antagonists inhibited the proliferative effects of IL-17, TGF-β2, and macrophage-conditioned medium, indicating a key role of GH-RH in the inflammatory pathways^93^. To gain an understanding of the role of GH-RH in inflammation-induced prostatic enlargement, we tested the efficacy of GH-RH antagonists in an advanced prostatitis model of carrageenan-induced chronic prostatitis (3). GH-RH antagonists reduce prostatic enlargement and inflammation in carrageenan-induced chronic prostatitis^95^. Inflammation plays a key role in the etiology of BPH through multiple pathways involving the stimulation of proliferation by cytokines and growth factors, as well as the induction of EMT.

Previously, we reported that GH-RH acts as a prostatic growth factor in experimental BPH and in autoimmune prostatitis models and its blockade with GH-RH antagonists offers therapeutic approaches for these conditions. In another study, we aimed to investigate the beneficial effects of GH-RH antagonists in λ-carrageenan-induced chronic prostatitis and probe the downstream molecular pathways implicated in GH-RH signaling^95^. Thus, 50 µL of 3% carrageenan was injected two times into both ventral prostate lobes of Sprague-Dawley rats, and a powerful GH-RH antagonist, MIA-690, was administered 5 days after the second intraprostatic injection at 20 µg daily dose for 4 weeks. GH-RH-induced signaling events were identified in BPH-1 and primary prostate epithelial cells at 5, 15, 30, and 60 min with Western blot. Inflammation-induced prostatic enlargement increased the area of the stromal compartment, whereas treatment with the GH- RH antagonist significantly reduced these effects^95^. This beneficial activity was consistent with a decrease in prostatic GH-RH, inflammatory marker COX-2, growth factor IGF-1, protein level of inflammatory and EMT marker TGF-β1, and the expression of multiple genes related to EMT. In vitro, GH-RH stimulated multiple pathways involved in inflammation and growth in both BPH-1 and PrEp cells, including NF-κB p65, AKT, ERK1/2, EGFR, STAT3, and increased the levels of TGF-β1 and the transactivation of the IGF receptor. This study demonstrated that GH-RH antagonists could be beneficial for the treatment of prostatic inflammation and BPH in part by inhibiting the growth-promoting and inflammatory effects of locally produced GH-RH^95^.

In another experimental study on BPH, we demonstrated shrinkage of experimental BPH and reduction of prostatic cell volume by a gastrin-releasing peptide (GRP) antagonist^96^. GRP is a potent growth factor in many malignancies. We have demonstrated that potent antagonists of GRP inhibit the growth of experimental human tumors including prostate cancer, and their effect on models of BPH has been studied. We then evaluated the effects of GRP antagonist RC-3940-11 on viability and cell volume of BPH-1 human prostate epithelial cells and WPMY-1 prostate stromal cells in vitro, and in testosterone-induced BPH in Wistar rats in vivo^96^. RC-3940-11 inhibited the proliferation of BPH-1 and WPMY-1 cells in a dose-dependent manner and reduced prostatic cell volume in vitro. Shrinkage of prostates was observed after 6 wks. of treatment with RC-3940-11: a 15.9% decline with 25 µg/day; and a 18.4% reduction with 50 µg/day. Significant reduction in levels of NF-κB /p50, cyclooxygenase-2, and androgen receptor was also seen. Analysis of transcript levels of genes related to growth, inflammatory processes, and signal transduction showed significant changes in the expression of more than 90 genes.

Together, GRP antagonists reduced the volume of human prostatic cells and lowered prostate weight in experimental BPH through direct inhibitory effects on prostatic GRP receptors. GRP antagonists should also be considered for further development as a therapy for BPH (73). Bombesin antagonists could be tried in combination with GH-RH antagonists in Wistar rats with Testosterone-induced BPH to determine the efficacy of this combination on BPH^96^.

## Conclusion

**Fig. 2:**
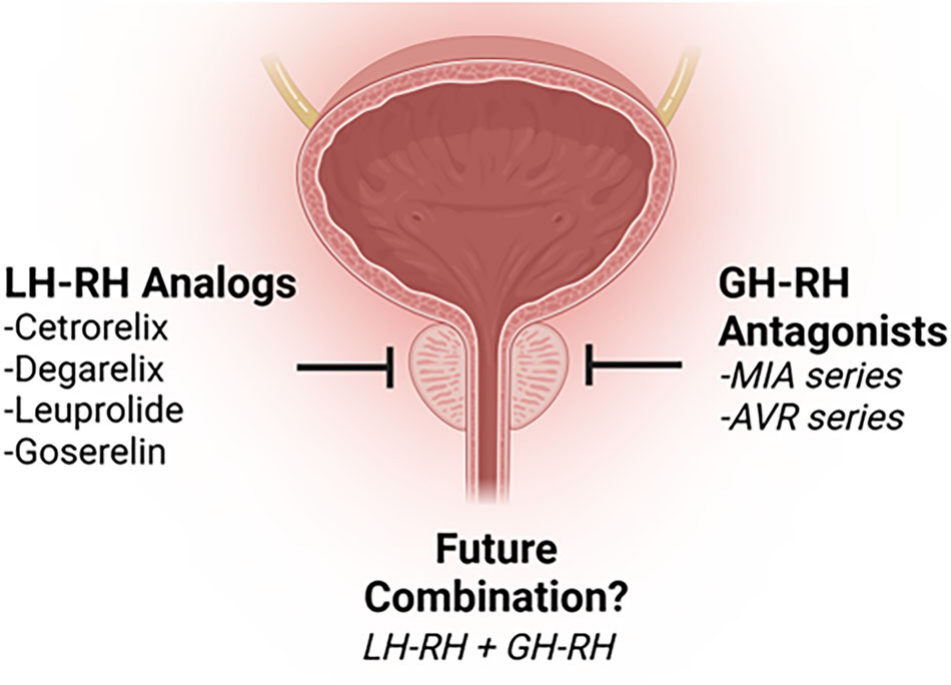
Therapeutic scheme for GH-RH and LH-RH analogs in blocking BPH development. The integration of GH-RH and LH-RH analogs into clinical practice could revolutionize BPH management, providing an effective, non-surgical alternative for patients with progressive disease.

Significant progress has been made globally by various investigators, laboratories, and pharmaceutical companies in understanding the hormonal regulation of BPH, with LH-RH and GH-RH analogs emerging as promising therapeutic options (Fig. 2 and Box 3). While LH-RH analogs have been widely used in prostate cancer and studied in BPH treatment by reducing androgen levels, GH-RH antagonists offer a distinct mechanism by inhibiting growth factors, inflammatory pathways, and cellular signaling involved in prostate enlargement. This review supports the concept that peptides could be effectively developed and delivered in the body (Box 3. 2000s), and that GH-RH antagonists significantly reduced the volume of prostatic cells and lowered prostate weight in experimental BPH (Box 3. 2010s and 2020 s). These effects were achieved through direct inhibitory actions on prostatic GH-RH receptors, as evidenced by significant tissue-specific reductions in levels of NF-κB/p50, cyclooxygenase-2, and androgen receptors, and changes in the expression of over 90 genes related to growth, inflammation, and signal transduction. The observed results reinforce the potential of GH-RH antagonists as a viable therapeutic option for BPH, particularly when used in combination with other treatments and perhaps with LH-RH antagonists (Fig. 2).

Box 3 Historical timeline and clinical implications of GH-RH receptors and antagonists

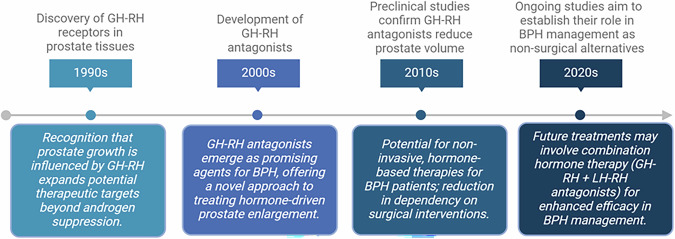

## Future directions

The promising outcomes from this research suggest several avenues for future investigation of the biology of BPH, LH-RH, and GH-RH in aging groups. Data from us and others support the application of GH-RH antagonists combined with other therapies as a simple rationale for investigation in clinical settings of BPH. Expanding on this rationale, further studies should evaluate the impact of combining GH-RH antagonists with other therapeutic agents, such as LH-RH antagonists and bombesin antagonists on BPH. This approach could potentially enhance therapeutic benefits in BPH and reduce the prostate volume more effectively than monotherapy (Fig. 2).

Further details into the molecular mechanisms of GH-RH antagonist actions and effects could be elucidated via mechanistic studies. Additional research should delve deeper into the molecular mechanisms by which GH-RH antagonists exert their antiproliferative and anti-inflammatory effects. This includes understanding the signaling pathways involved and identifying biomarkers that can predict treatment response in the aged. With the common availability of vastly improved molecular testing/ molecular pathology, not available when this work started 50 years ago, we can test molecules that may be associated with improved treatment outcomes. Surely, collection and analysis of tissues may also provide more detail of the tissue-specific molecular effects of these peptides as well. This line of research could also identify additional molecules that may be targeted by existing or new agents in these signaling paths.

Given the positive preclinical results and the growth of our aging populations, it is imperative to advance GH-RH antagonists into clinical trials to improve BPH outcomes. This direction will help determine safety, optimal dosing, and efficacy and capture any potential side effects and safety signals through comprehensive toxicological studies. The translational potential of these antagonists depends on such clinical studies, and the continued rising global prevalence of BPH warrants these investigational efforts.

We also propose expanded applications of GH-RH antagonists by investigating their potential use in other hormone-related conditions and malignancies like lung disease. Another disease that is affecting our aging population at large and the aging Veterans. Tissue and indication-related specificity may be found with studies of lung cancer and lung fibrosis. The ability of antagonists to inhibit growth and inflammation suggests that they could be beneficial in treating these other disorders characterized by similar pathophysiological mechanisms.

Our efforts always include a special focus on US Service Members since most of our work has been conducted in the setting of the US Department of Veterans Affairs. Studies among this population are warranted due to the high prevalence of BPH among service members, military personnel, and aging Veterans. Hence, we propose these studies within the VA healthcare system, the largest integrated healthcare system in the US, that could provide valuable insights and directly benefit this patient population and the aging population at large.

## Abbreviations

BPH: Benign prostatic hyperplasia
COS-ART: controlled ovarian stimulation/assisted reproductive technology
CRPC: castration-resistant prostatic cancer
COX-2: cyclooxygenase-2
EGF: Epidermal growth factor
EMT: epithelial-to-mesenchymal transition
FSH: follicle-stimulating hormone
GH-RH: growth hormone-releasing hormone
GRP: gastrin-releasing peptide
GLY: glycine
Gn-RH: Gonadotropin Releasing Hormone
IGF-I: insulin-like growth factor-1
IVF-ET: in vitro fertilization and embryo transfer
LEU: Leucine
LH-RH: luteinizing hormone-releasing hormone
MEF: mouse embryonic fibroblasts
pGHRH-R: pituitary-GH-RH receptor
PHE: phenylalanine
PSA: prostate-specific antigen
TRP: tryptophan
TURP: transurethral resection of the prostate
